# Differences in Cognition and Smoking Abstinence Rates Among People With and Without HIV Who Smoke

**DOI:** 10.1093/ntr/ntaf115

**Published:** 2025-06-27

**Authors:** Sera Levy, Robert A Schnoll, E Paul Wileyto, Morgan Thompson, Manaal Azad, David Metzger, Frank Leone, Rebecca L Ashare

**Affiliations:** Department of Psychology, State University of New York at Buffalo, Buffalo, NY, USA; Department of Psychiatry, Perelman School of Medicine, University of Pennsylvania, Philadelphia, PA, USA; Department of Biostatistics, Epidemiology, and Informatics, Perelman School of Medicine, University of Pennsylvania, Philadelphia, PA, USA; Department of Psychiatry, Perelman School of Medicine, University of Pennsylvania, Philadelphia, PA, USA; Department of Psychology, State University of New York at Buffalo, Buffalo, NY, USA; Department of Psychiatry, Perelman School of Medicine, University of Pennsylvania, Philadelphia, PA, USA; Pulmonary, Allergy, and Critical Care Division, Department of Medicine, University of Pennsylvania, Philadelphia, PA, USA; Department of Psychology, State University of New York at Buffalo, Buffalo, NY, USA

## Abstract

**Introduction:**

High rates of smoking among people with HIV (PWH) persist and may be due to HIV-associated neurocognitive disorders exacerbating abstinence-induced cognitive deficits, leading to higher risk of relapse. This study assessed differences in smoking abstinence rates and abstinence-induced cognitive deficits among PWH and people without (PWOH).

**Methods:**

In this prospective observational design (NCT03169101), treatment-seeking adults completed two laboratory sessions during a pre-quit phase to assess cognition: once following 24h abstinence and once smoking-as-usual. Cognition was measured through response inhibition, working memory, and verbal memory tasks. All received standard smoking cessation treatment over 8 weeks (i.e., counseling, nicotine patch). Point-prevalence abstinence was assessed at end-of-treatment.

**Results:**

Our sample included 210 participants (38.1% PWH; 61.9% PWOH), who were mostly male (59.5%) and Black/African-American (76.7%). No significant HIV status by abstinence condition interactions emerged for any cognitive outcome (all *p*s > .4). There were significant abstinence-induced deficits in response inhibition (*p* = .02), working memory response time (*p* = .005), and verbal memory (*p*=<.001). No significant differences emerged in abstinence rates between PWH and PWOH (31.2%, 32.3%, respectively; OR = 1.26, 95% CI: 0.67, 2.39, *p* = .48).

**Conclusion:**

Despite prior research suggesting differences in abstinence rates and cognition between PWH and PWOH who smoke, hypotheses were not supported. However, this is one of a few studies to directly compare people with and without HIV in a rigorously designed mechanistic smoking cessation study. Given that cognition does not appear to play a primary role in smoking among PWH, more work is needed to understand the mechanisms driving disproportionate smoking rates among PWH.

**Implications**

Findings from this prospective, observational, clinical trial suggest that abstinence-induced cognitive deficits do not differ among people with HIV (PWH), as compared to people without (PWOH), and further, cognitive deficits among PWH did not mediate end of study abstinence. This work adds to the field by directly comparing PWH and PWOH to explore a potential mechanism that may explain heightened rates of smoking among PWH, though evidence for this was not found.

## Introduction

Since the introduction and widespread availability of antiretroviral therapies (ART) for the management of human immunodeficiency virus (HIV), the life expectancy gap has narrowed between individuals living with HIV (PWH) and individuals living without.^1,2^ Recent epidemiological data between 2011 and 2016 report that PWH on ART with no comorbidities have a life expectancy of only 5–8.5 years less than individuals without HIV, which is a drastic improvement compared to data from 2000 to 2003 that revealed a difference of 20–24 years.^3^ Unfortunately, components outside of HIV-related factors such as comorbid Hepatitis B or C, alcohol/drug use, and tobacco use likely contribute to why a life expectancy gap persists.^4^ Additionally, PWH have a higher risk of developing cardiovascular diseases, so efforts to combat modifiable risk factors for both HIV-related mortality and cardiovascular disease, like smoking, is of particular importance.^5^

Despite a general decrease in smoking within the general population, higher rates of smoking among PWH remain.^6^ A 2021 meta-analysis estimated that PWH were 1.64 times more likely to report smoking as compared to people without HIV (PWOH), suggesting that this may be a driver of the maintained life expectancy gap.^7^ Indeed, PWH who smoke experience a greater number of life-years lost attributable to smoking than to any HIV-related ailments.^8^ In fact, even PWH who have good ART adherence are more likely to die from lung cancer than from HIV-related causes if they report smoking, suggesting that smoking may outweigh the beneficial effects of ART.^9^

One potential consequence of tobacco use among PWH is the effect of smoking on HIV pathogenesis, possibly leading to accelerated incidence and progression of HIV-associated neurocognitive disorders (HANDs).^10^ Tobacco use among the general population has shown an association with a greater risk for neurocognitive deficits.^11,12^ On the other hand, research has also highlighted the cognitive-enhancing effects of nicotine, which appears to play a relevant role in relapse after experiencing tobacco withdrawal.^13^ Therefore, individuals experiencing nicotine withdrawal-related cognitive deficits might be more inclined to resume smoking to ameliorate these effects, resulting in an operant conditioning cycle of continuing to smoke to prevent unwanted cognitive deficits.^14–16^ Indeed, cognitive deficits experienced during the withdrawal period have been shown to predict relapse.^17^

Approximately 25%–46% of PWH experience a degree of cognitive deficits related generally to HANDs; however, one study identified that current smoking negatively impacted memory, verbal fluency, processing speed, and executive functioning among PWH.^18–20^ Despite prior research linking smoking withdrawal-induced cognitive deficits with relapse,^15,16^ in addition to higher smoking prevalence among PWH and cognitive deficits, no study has assessed whether withdrawal-associated cognitive deficits are risk factors for relapse among PWH. Thus, it is possible that the combination of cognitive deficits associated with HANDs and the cognitive deficits associated with smoking abstinence make smoking cessation more difficult for PWH. However, this research question has not been explored until now.

The present study sought to examine if abstinence-induced cognitive deficits serve as a primary mechanism that may underlie smoking persistence among PWH. We hypothesized that PWH would have greater abstinence-induced cognitive deficits compared to PWOH and lower abstinence rates. We also hypothesized that these differences would predict relapse at the end of treatment and that this would mediate the differences in abstinence rates between PWH and PWOH.

## Material and Methods

### Study Design

This was a prospective observational design that was divided into two phases: a pre-quit laboratory phase (weeks 0–2) and a treatment phase (weeks 3–12). The trial was registered with ClinicalTrials.gov (NCT03169101), approved by the University of Pennsylvania IRB, and conformed to US Federal Policy for the Protection of Human Subjects. Written informed consent was obtained from participants.

### Participants

Participants were recruited through the University of Pennsylvania’s Infectious Diseases Division, community-based HIV clinics, and advertisements. Participants were eligible if they were at least 18 years old, self-reported smoking at least 5 cigarettes per day, able to use transdermal nicotine (TN) safely (confirmed via medical evaluation), and capable of communicating in English. PWH had to be treated with ART and document viral load ≤1000 copies/mL and CD4 + counts > 200 cell/mm3 within 12 months of enrollment. PWOH had to document negative HIV status or complete on-site rapid HIV test at intake. Other exclusion criteria included current use of anti-psychotic medications and/or diagnosis of psychotic disorder, unstable and untreated major depression (e.g., current suicidal ideation), self-reported current or planned pregnancy, self-reported current use of smoking cessation medications, and uncontrolled hypertension. Participants who had a positive urine drug screen at Intake for cocaine, methamphetamines, PCP, barbiturates, or ecstasy were deemed ineligible. Participants in current treatment for substance use disorder were reviewed on a case-by-case basis by the study physician.

### Procedures

Recruited individuals completed an initial eligibility assessment either in the clinic or over the phone prior to the Intake Session, during which the consenting process was completed, and eligibility was determined. Once enrolled, participants completed two sessions during the pre-quit period during which cognitive tasks were administered: one session after 24 hours of smoking abstinence and one session while smoking-as-usual. The order of sessions was randomized between participants and counter balanced. The first laboratory session was scheduled within 1–2 weeks of the Intake Session and the second laboratory session took place 1 week after the first. During these sessions, participants provided urine specimens for drug and pregnancy screening, a carbon monoxide (CO) breath sample, and had blood pressure assessed. Additionally, they completed questionnaires that assessed medications, adherence to ARTs, depression, anxiety, nicotine withdrawal, functional impairment, affect, subjective cognitive complaints, and smoking rate via timeline follow-back. Finally, participants completed the cognitive tasks. For the smoking-as-usual condition, participants smoked a cigarette in a specially ventilated room in the lab approximately 30–40 minutes before the cognitive task battery. For the abstinent condition, participants needed to have a CO reading of less than 10ppm or less than 50% CO reading taken at Intake to be eligible. Additionally, participants were instructed to not use any form of nicotine replacement therapy during their 24-hour abstinence period. If participants either reported smoking in the last 24 hours or did not meet CO criteria during the abstinent session, study staff rescheduled the appointment.

Following the two-week pre-quit phase, an 8-week treatment phase began in which all participants received standard smoking cessation treatment, including brief individual counseling (weeks 3–8) and eight weeks of open-label TN treatment (weeks 4–12). The counseling followed PHS guidelines and was delivered by trained counselors who were supervised by a clinical psychologist.^21^ The behavioral counseling began at week 3 with a pre-quit session, during which a quit plan was developed to identify and manage triggers.^22^ Additionally, the counselor explained the role of the TN patch in withdrawal symptom management and how to use the patch. For PWH, one HIV-specific module was added to provide education about the unique health risks associated with smoking among PWH.^23^ During week 4 (Target Quit Date), participants received a 30-minute session to discuss the initial quit attempt, identify possible reasons for relapse, and review a plan to avoid future tempting situations. TN patch adherence was also emphasized. During weeks 5 to 8, participants received four 20-minute relapse prevention sessions during which the counselor would reinforce success and review the quit plan, or help the participant reestablish a quit date. All sessions were recorded and 15% were assessed for protocol adherence by senior study staff.

Participants began an 8-week TN regimen the morning of the target quit date (i.e., week 4), and were instructed to use the TN patches in a tapering fashion as recommended by the manufacturer (e.g., participants who smoke 10 or more cigarettes per day would use 21mg patches for 4 weeks, 14mg for 2 weeks, and 7mg for 2 weeks).

At week 12, participants completed an end-of-treatment (EOT) follow-up visit in which biochemical verification of self-reported abstinence was conducted, in addition to completing self-report smoking assessments, CO, and additional self-report questionnaires (i.e., to assess anxiety, depression, nicotine withdrawal, etc.).

### Cognitive Measures

All cognitive measures were presented via E-Prime 2.0 (Psychology Software Tools, Inc) unless otherwise noted. Executive functioning was measured by use of the Stroop test and a working memory N-Back task. In the Stroop test,^24^ participants were shown a series of words and asked to press the key associated with the color of the word and not the word itself. Congruent trials are the trials in which the color and word match (e.g., the word “green” appears in the color green), and incongruent trials display words printed in colors that do not match the word (e.g., the word “green” appears in the color blue). An interference score (primary outcome) is calculated by subtracting the reaction time to congruent trials from the reaction time to incongruent trials. For the working memory N-Back task, participants were presented with sequences of fractal images and used a button press to a choose a single target using the following rules: in the 1-back condition, participants responded if the image was identical to the one preceding it; in the 2- and 3-back conditions, they responded if the image was identical to the one two and 3 trials before, respectively.^25^ The active baseline condition (0-back) is a simple target detection task. Hit rate (percent correct responses) and correct reaction time were assessed as primary outcomes.

Response inhibition was assessed through the Stop Signal Task (SST), which has been used in prior work with people who smoke and is sensitive to abstinence.^26,27^ Participants were instructed to respond to left- and right-facing arrows on the computer screen. After several practice trials, participants completed three 64-trial task blocks with stop signals (100-ms tone) presented on 25% of the trials. The baseline stop delay in each block was 250ms but was adjusted ± 50ms depending on whether the participant successfully inhibited. Trials consisted of a 500-ms warning stimulus, a 1000-ms go signal (left- and right-facing arrows), and a 1000-ms blank screen inter-trial interval. The primary outcome from this task was a stop signal reaction time, which was calculated by subtracting the mean stop delay (MSD) from the mean reaction time on the go-trials (MRT).

Verbal learning and memory were measured using the Hopkins Verbal Learning Test—Revised (HVLT-R), in which immediate recall, delayed recall, and delayed recognition were assessed.^28^ The version used in this trial had six alternate forms with each form containing 12 nouns and four words from one of three semantic categories. Participants were shown words over the course of three learning trials and given a delayed recall and recognition trial approximately 25 minutes after learning. The HVLT-R has high test-retest reliability and its concurrent, discriminant, and construct validity have been well-established.^29^ The two primary outcomes obtained from the HVLT-R were Total Recall and Delayed Recall T-scores.

### Smoking Outcomes

Self-reported smoking was assessed via timeline follow-back. Self-reported abstinence was confirmed biochemically using a carbon monoxide (CO) breath sample. The primary outcome was 7-day point-prevalence abstinence at EOT defined as self-reported abstinence for at least 7 days prior to EOT and a CO of less than 5 ppm.^30,31^ CO was measured at all study visits.

### Covariate Measures

Demographic data (e.g., age, gender, marital status, and education) and smoking history (e.g., age of smoking initiation, cigarette brand, length of prior abstinence periods, and current smoking rate) were collected at intake. Nicotine dependence was assessed by the Fagerstrom Test for Nicotine Dependence (FTND)^32^ which is a 6-item self-report measure that has demonstrated high test-retest reliability (*r* = 0.88).^33^ The Shipley Institute of Living Scale (SILS) was administered during the Intake Session to assess general intellectual functioning. The SILS is a self-report test that has been validated for identifying cognitive impairments.^34^ The SILS yields an overall score which correlates with the Wechsler Adult Intelligence Scale-Revised (WAIS-R) Estimated IQ Test.^34^

### Data Analysis

Preliminary analyses were conducted to determine whether participant characteristics (e.g., gender, nicotine dependence) were associated with HIV status or outcomes using either chi-square, t-tests, or correlations. Variables showing potential to predict outcome (*p* = .2) were tested for entry as controlling variables. Covariates included race, sex, nicotine dependence (FTND score), age, income, and SILS. For the cognitive tasks, the top and bottom 5% of scores were Winsorized to reduce the impact of outliers. An interaction effect was tested between HIV status and abstinence condition (i.e., smoking or abstinent) across each cognitive outcome. Abstinence condition order was also included to control for order effects. Primary analyses include participants who completed the laboratory phase and began treatment. Following an intent-to-treat protocol, participants who were lost to follow-up, had missing data, or missed biochemical verification were treated as smoking. We also conducted a completers only analysis, to examine the sensitivity of the primary findings to our assumptions. Effects of abstinence on cognitive measures were assessed via multiple linear regression using a GLM framework with abstinence condition (abstinent vs smoking-as-usual) as a within-subject factor and HIV status as a between-subject factor. Point-prevalence abstinence was assessed via logistic regression. All analyses were conducted with STATA (StataCorp, College Station, TX).

## Results

### Patient Characteristics

**Figure 1.**
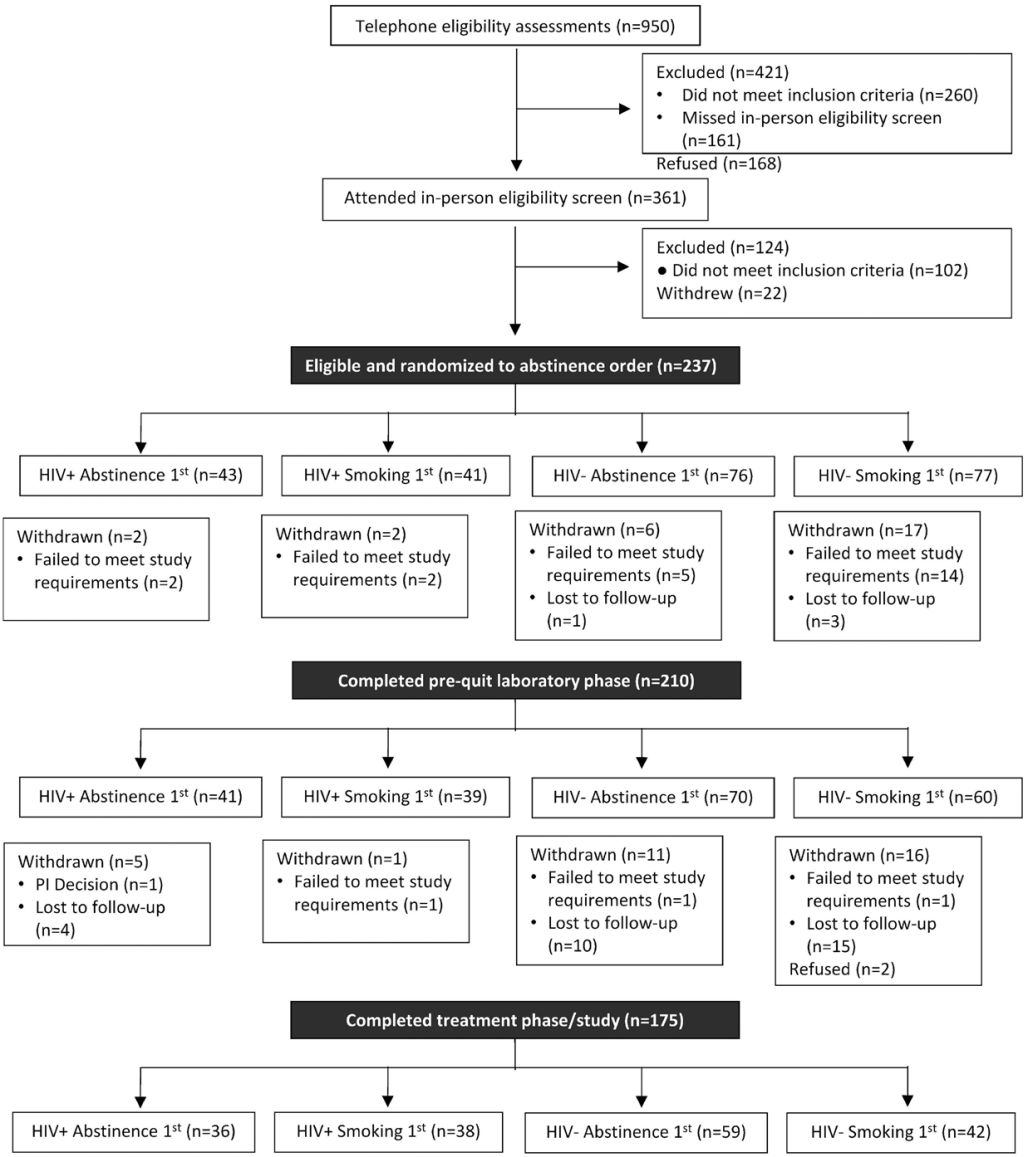
CONSORT diagram depicting participant flow throughout study.

The study was conducted from October 2016 through April 2022. As shown in the CONSORT diagram (Figure 1), 237 participants were randomized to an abstinence order condition and 210 completed the pre-quit laboratory phase (primary sample analyzed): 111 to abstinent and 99 to smoking as usual first. Approximately 61% were male, 73% were Black/African-American, and 79% indicated that they had at least a high school education (Table 1). PWH were more likely to identify as Black/African-American and have lower income (*p* = .002) than PWOH. Otherwise, there were no significant differences by HIV status or lab session order. Overall, 175 participants completed the study (74 PWH and 101 PWOH). Analyses were conducted to assess for characteristics associated with attrition. PWOH (vs PWH) and those who were randomized to the abstinent condition first (vs smoking condition first) were more likely to be lost-to-follow up (34% vs 12% *p < *.001; 80% vs. 68%; *p = *.035, respectively). Other characteristics were not associated with attrition.

**Table 1.** Participant Characteristics by HIV Status and Session Order

	HIV status by lab session order						
	PWH abstinent 1st	PWH smoking 1st	PWOH abstinent 1st	PWOH smoking 1st	Total	HIV status *p*-value	Lab Session order *p*-value
*N*	41 (19.5%)	39 (18.6%)	70 (33.3%)	60 (28.6%)	210		
Age	50.9 (10.0)	53.4 (9.6)	51.5 (11.1)	50.1 (12.3)	51.3 (11.0)	.42	.92
Sex							
Male (*n*,%)	28 (68.3%)	26 (66.7%)	43 (61.4%)	28 (46.7%)	125 (59.5%)	.07	.17
Race							
Asian	0 (0.0%)	1 (2.6%)	1 (1.4%)	0 (0.0%)	2 (1.0%)	.002	.57
Black/ African-American	38 (92.7%)	34 (87.2%)	45 (64.3%)	44 (73.3%)	161 (76.7%)		
Native Hawaiian/ Pac. Isl.	0 (0.0%)	0 (0.0%)	1 (1.4%)	0 (0.0%)	1 (0.5%)		
White	1 (2.4%)	2 (5.1%)	17 (24.3%)	9 (15.0%)	29 (13.8%)		
More than one race	1 (2.4%)	1 (2.6%)	1 (1.4%)	2 (3.3%)	5 (2.4%)		
Unknown or not reported	1 (2.4%)	1 (2.6%)	4 (5.7%)	5 (8.3%)	11 (5.2%)		
Refused	0 (0.0%)	0 (0.0%)	1 (1.4%)	0 (0.0%)	1 (0.5%)		
Education							
Grade school	1 (2.4%)	2 (5.1%)	1 (1.4%)	0 (0.0%)	4 (1.9%)	.33	.99
Some high school	8 (19.5%)	8 (20.5%)	12 (17.1%)	9 (15.0%)	37 (17.6%)		
HS grad or GED	14 (34.1%)	12 (30.8%)	19 (27.1%)	20 (33.3%)	65 (31.0%)		
Some college/ technical school	15 (36.6%)	13 (33.3%)	27 (38.6%)	22 (36.7%)	77 (36.7%)		
College grad or beyond	3 (7.3%)	4 (10.3%)	11 (15.7%)	9 (15.0%)	27 (12.9%)		
Income (above/below $20K)							
< $20,000 (*n*, %)	31 (75.6%)	25 (65.8%)	33 (47.8%)	30 (50.0%)	119 (57.2%)	.002	.76
Shipley Institute of Living Scale	85.1 (14.0)	91.9 (12.1)	91.6 (14.7)	88.7 (13.0)	89.6 (13.8)	.34	.68
Viral load baseline							
Undetectable (*n*, %)	37 (92.5%)	34 (89.5%)	–	–	71 (91.0%)	.640	–
Past/stable current major depressive episode							
Yes (*n*, %)	10 (25.0%)	5 (13.2%)	9 (12.9%)	6 (10.3%)	30 (14.6%)	.14	.24
CPD	10.6 (5.3)	12.5 (5.4)	13.8 (6.1)	12.3 (6.0)	12.5 (5.9)	.06	.81
FTND score	4.6 (2.0)	5.1 (1.9)	4.9 (2.0)	4.9 (2.0)	4.9 (2.0)	.77	.45

CPD = cigarettes per day; FTND = Fagerstrom test for nicotine dependence. * = Significant difference for HIV status, *p* = .002.

### Abstinence-Induced Cognitive Deficits Across PWH and PWOH

No interaction effects emerged as significant (all *p*s > .3; Table 2). We then evaluated main effects of both HIV status and abstinence, in which a significant main effect of HIV status on response inhibition (*b = *−19.9, 95% CI: −38.4, −1.45, *p = *.04) emerged. Patterns were similar for working memory accuracy (*b = *0.032, 95% CI: −0.004, 0.069, *p = *.09) and working memory reaction time (*b = *−28.6, 95% CI: −58.8, 1.58, *p = *.06), but they did not reach statistical significance. Specifically, PWH had marginally higher stop signal reaction time (i.e., worse response inhibition) and worse accuracy and slower response time on the N-back task. Additionally, there were significant main effects of abstinence for response inhibition (*b = *11.0, 95% CI: 1.69, 20.3, *p* = .02), working memory response time (*b = *15.7, 95% CI: 4.31, 27.1, *p = *.007), and verbal memory (*b = *−2.66, 95% CI: −4.09, −1.22, *p < *.001). This indicated that response inhibition, working memory response time, and verbal memory were worse during abstinence, compared to the smoking as usual session across PWH and PWOH (see Table 3). Holm’s method was utilized to adjust *p*-values for multiple comparisons, in which only the delayed recall task remained significant at the *p* < .05 level.^35^

**Table 2.** Regression Models Predicting Cognitive Function by HIV Status and Smoking Condition

	Stop Signal Reaction Time	Delayed Recall	Working Memory: Accuracy	Working Memory: RT (ms)	Stroop Interference (ms)
HIV status (PWOH = ref) PWH					
Coefficient	22.77	−0.86	−0.03	27.73	29.02
95% CI	[2.04, 43.49]	[−3.79, 2.07]	[−0.06, 0.01]	[−4.63, 60.09]	[−8.72, 66.76]
*p*-value	0.031	0.566	0.206	0.093	0.132
Smoking condition (smoking as usual = ref) Abstinent					
Coefficient	13.19	−2.36	0.01	15.03	−2.20
95% CI	[1.42, 24.95]	[−4.20, −0.53]	[−0.01, 0.03]	[0.47, 29.59]	[−30.41, 26.00]
*p*-value	0.028	0.012	0.361	0.043	0.878
HIV status × smoking condition					
Coefficient	−5.84	−0.76	−0.01	1.75	−10.24
95% CI	[−25.12, 13.45]	[−3.71, 2.19]	[−0.04, 0.02]	[−21.63, 25.12]	[−55.44, 34.97]
*p*-value	0.553	0.614	0.373	0.884	0.657
Lab session number (first = ref) Second lab session					
Coefficient	−11.61	−1.61	0.04	6.01	−35.45
95% CI	[−20.93, −2.29]	[−3.05, −0.17]	[0.03, 0.06]	[−5.40, 17.41]	[−57.52, −13.38]
*p*-value	0.015	0.029	0.000	0.302	0.002
Race (White = ref) Black/African-American					
Coefficient	−7.25	−2.61	0.02	−2.01	20.60
95% CI	[−33.93, 19.42]	[−6.23, 1.02]	[−0.04, 0.07]	[−45.27, 41.26]	[−23.01, 64.20]
*p*-value	0.594	0.159	0.544	0.928	0.355
Race (All others)					
Coefficient	−24.91	−5.21	−0.01	−28.41	22.13
95% CI	[−60.10, 10.28]	[−10.10, −0.32]	[−0.08, 0.07]	[−86.78, 29.96]	[−36.39, 80.65]
*p*-value	0.165	0.037	0.885	0.340	0.459
Gender (Male = ref) Female					
Coefficient	6.85	2.79	0.03	−3.78	9.61
95% CI	[−10.44, 24.14]	[0.42, 5.15]	[−0.01, 0.06]	[−32.05, 24.50]	[−18.80, 38.01]
*p*-value	0.437	0.021	0.097	0.793	0.507
Nicotine dependence (FTND)					
Coefficient	1.34	−0.23	−0.00	1.86	2.52
95% CI	[−3.03, 5.72]	[−0.83, 0.38]	[−0.01, 0.01]	[−5.32, 9.03]	[−4.68, 9.73]
*p*-value	0.548	0.463	0.879	0.612	0.492
Age (years)					
Coefficient	1.16	−0.15	−0.00	1.69	1.25
95% CI	[0.37, 1.96]	[−0.26, −0.04]	[−0.00, −0.00]	[0.37, 3.00]	[−0.08, 2.57]
*p*-value	0.004	0.006	0.000	0.012	0.065
SILS					
Coefficient	−0.32	0.32	0.00	1.44	1.13
95% CI	[−0.98, 0.33]	[0.23, 0.41]	[0.00, 0.01]	[0.37, 2.51]	[0.06, 2.20]
*p*-value	0.332	0.000	0.000	0.008	0.038
Income (<$20,000 = ref) >=$20,000					
Coefficient	−16.79	0.26	0.03	2.50	−31.64
95% CI	[−34.55, 0.97]	[−2.17, 2.68]	[−0.00, 0.07]	[−26.48, 31.48]	[−60.70, −2.58]
*p*-value	0.064	0.836	0.086	0.866	0.033

**Table 3.** Mean (SEM) for Cognitive Tasks by HIV Status and Smoking Condition

	PWOH abstinent		PWOH smoking		PWH Abstinent		PWH smoking	
	Mean	SEM	Mean	SEM	Mean	SEM	Mean	SEM
Stop signal response time (ms)	304.8	5.97	293.8	5.97	319.8	7.5	308.8	7.4
Verbal memory (Delayed recall)	33.5	0.84	36.1	0.84	32.0	1.03	34.6	1.03
Working memory (accuracy, % correct)	0.643	0.012	0.635	0.012	0.605	0.015	0.609	0.015
Working memory (response time, ms)	623.4	9.89	608.4	9.88	652.9	12.64	636.14	12.64
Stroop interference score (ms)	120.4	11.69	122.6	11.68	139.2	14.87	151.6	14.80

### Probability of Quitting After Standard Treatment Across PWH and PWOH

The overall abstinence rate was 32%, with no difference by HIV status (31.2% in PWH and 32.3% in PWOH), including in a logistic regression analysis using PWOH as the referent group, and race, age, sex, and nicotine dependence as covariates (OR = 0.79, 95% CI: 0.67, 2.39, *p* = .48). Due to the missing data, we conducted an exploratory multiple imputation analysis, and the results were unchanged. In the completers-only analysis (*n* = 175), abstinence rates across HIV status remained nonsignificant (33.8% in PWH and 40.6% in PWOH), but the pattern was more consistent with our hypothesis (OR = 0.63, 95% CI: 0.83, 3.2, *p* = .16).

### Mediation of Abstinence-Induced Cognitive Deficits on Smoking Relapse Between PWH and PWOH

To assess this question, the abstinence effect on cognitive deficits was estimated by calculating the difference in total scores on the response inhibition, working memory (accuracy and response time), and verbal memory during the smoking-as-usual cognitive task session and the abstinence session. Logistic regression models were utilized to assess if the abstinence effect predicted 7-day point-prevalence abstinence and whether this differentially predicted abstinence by HIV status (HIV × abstinence effect interaction). No significant findings emerged for the abstinence effect or interaction with HIV status for any of the cognitive outcomes, indicating that relapse was not mediated by abstinence effects on cognitive deficits.

## Discussion

This study examined the abstinence-induced cognitive profiles and relapse rates across PWH and PWOH enrolled in a smoking cessation study. Participants completed two cognitive testing sessions, one after a 24-hour abstinence and one after a smoking-as-usual period, with a balanced order randomized between participants. After the cognitive testing phase, participants were given standardized smoking cessation treatment, and smoking status was assessed at end of treatment. It was hypothesized that abstinence-induced cognitive deficits would be greatest among PWH, that these deficits would predict relapse at the end of treatment, and that this would mediate the differences in abstinence rates between PWH and PWOH who smoke; however, the analyses did not support these hypotheses. Instead, abstinence effects on cognition emerged across both PWH and PWOH groups including response inhibition, working memory response time, and verbal memory. This is consistent with other research that suggests acute abstinence from smoking disrupts the neurophysiological index of general performance, which is involved in a range of cognitive functions.^14^ Additionally, no significant differences emerged in relapse rates between PWH and PWOH. Though somewhat surprising, this finding is consistent with another study that assessed abstinence rates following a quit attempt.^36^

Across both laboratory sessions, compared to PWOH, PWH in our sample had worse response inhibition and although not significant, there were similar patterns for working memory accuracy and response time. This is consistent with the current state of knowledge on HAND, suggesting that there is a possible interplay among viral load, ART, and one’s cognitive reserve that may negatively impact cognition.^37,38^ Of relevance, PWH in our sample self-reported high adherence to their ART. Some research has proposed possible neurotoxicity from several types of ART medications, and some patients taking ART report cognitive side effects,^39^ while other studies show higher cognition among some groups currently on ART compared to those who are not.^37,40^ This may be due to ART improving overall well-being, as a meta-analysis concluded that ART may only improve cognition among PWH with poor physical conditions and immunity status, not necessarily the general population with HIV.^37^ Although all PWH in the current study were on stable ART regimens, we did not have power to evaluate differences in type of ART regimen. Similarly, because all the PWH in this study smoked tobacco, we cannot evaluate whether ART and tobacco use interact. These are questions for future studies.

Our data also suggested that there were no significant differences in the likelihood of being abstinent by HIV status. This finding is puzzling in consideration of the heightened prevalence of smoking in the PWH population compared to the general population, combined with data from smoking cessation clinical trials suggesting lower efficacy of treatments.^7,41^ Therefore, we predicted that relapse would be more common among PWH. Although few studies have directly compared abstinence rates between PWH and PWOH, there is some evidence suggesting no significant differences in six-month abstinence rates across PWH and PWOH^36,42^ and similar cessation rates among both PWH and PWOH in an environment with access to cessation resources.^43^ However, these prior studies were either retrospective analyses or pooled data across different studies. The current prospective study builds on this evidence because it was specifically designed to compare abstinence rates between PWH and PWOH. Of note, PWH in our study were more likely to complete the study, compared to PWOH. This is consistent with data suggesting a high rate of reported quit attempts among PWH and a higher likelihood of initiating smoking cessation treatment, compared to PWOH.^43^ Thus, more research is needed to better understand the high prevalence of smoking among PWH.

Perhaps other factors are relevant to understanding the heightened smoking rates among PWH, such as the presence of co-occurring mental illnesses and social determinants of health.^44^ The current study was precluded from testing the role of mental illness on abstinence because individuals with current psychiatric disorders were excluded and only ~15% of the sample reported past or stable current major depressive disorder. Additionally, PWH is a group that is subject to higher levels of stigma and discrimination, and prior research suggests that endorsed stigma and discrimination may be related to smoking.^45,46^ Misinformation about tobacco use may also be relevant, as one study found that 27% of their population of PWH thought (mistakenly) that smoking raised their T-cell counts and helped fight infections.^47^ Additionally, smoking behavior can be reinforced through cultural and societal norms, which may be a means for vulnerable groups to feel socially accepted and alleviate feelings of isolation and loneliness.^48^ Many factors, such as smoking for stress management, enjoyment of smoking, nicotine addiction, habit; access to quit resources; boredom, pro-smoking living environments, and socioeconomic disadvantage are barriers to quitting smoking.^49^ These factors may be important to study in future work.

Interestingly, the order of smoking condition for the lab sessions was significantly related to completing the study. Specifically, 80% of participants randomized to complete the abstinent session first completed the study compared to 68% of those who had the smoking session first. This is noteworthy for future smoking intervention research. Perhaps participants who completed an abstinence period first received the positive effects associated with “practice quits,” that is, having an individual go through a short cessation period prior to a quit date. One scoping review of practice quit attempts concluded that practice quit attempt groups tend to have better outcomes than the control.^50^ The hypothesized mechanism for practice quit attempts is akin to exposure therapy, in which exposure (without engaging in experiential avoidance) to withdrawal symptoms fosters habituation.^51^ Perhaps individuals in our study who partook in the abstinence period first experienced this habitation early in the study, which in turn, maintained their motivation. Thus, future research should consider these findings when designing studies to optimize retention.

Several limitations should be noted for this study. Our sample of PWH was smaller than the group of PWOH which may have limited power. In addition, there were differences in sociodemographic characteristics (income, race) between PWH and PWOH. Although we controlled for these variables in analyses, future studies should attempt to match groups on important risk factors. Moreover, lower retention rates among PWOH may have skewed the abstinence data (because missing data was coded as smoking). However, in our supplemental completers only analysis (see Supplementary Table S1), the point-prevalence abstinence rates were still not significantly different. Other limitations relate to the possible noncompliance of NRT patches during the treatment period. While participants were instructed to use these patches as directed, and self-reported high degree of compliance, we were unable to ensure this. Further, it is possible that act of being observed in the study may have influenced performance on the cognitive tasks, and this may not be an accurate reflection of cognition outside of the research setting.

## Conclusions

This study provides several important contributions to the HIV and smoking literature. Importantly, this is one of only a few studies that directly compared smoking abstinence in PWH to PWOH. We did not find differential abstinence-induced cognitive deficits or relapse rates between PWH and PWOH, suggesting that other mechanisms may be operating to influence the disparity in tobacco use among PWH. In addition, PWH were more likely to complete the study compared to PWOH, suggesting a strong level of engagement in the quitting process among this population. As such, more research is needed to identify the mechanism that is contributing to high smoking rates and decreased life expectancy in the HIV population so that targeted, evidence-based cessation interventions can be deployed.

## Supplementary Material

ntaf115_suppl_Supplementary_Table_S1

## Data Availability

Data is available upon request.
