# Supplementary material for: Differences in Cognition and Smoking Abstinence Rates Among People With and Without HIV Who Smoke
Source: Nicotine Tob Res. 2025 Jun 27;27(10):1831–9. doi: 10.1093/ntr/ntaf115 (PMC12453680; doi:10.1093/ntr/ntaf115)
Supplement: ntaf115_suppl_Supplementary_Table_S1 [file ntaf115_suppl_supplementary_table_s1.docx]

| **Completers-only Analysis** | | | | | |
| --- | --- | --- | --- | --- | --- |
|  | Stop Signal Reaction Time | Delayed Recall | Working Memory: Accuracy | Working Memory: RT (ms) | Stroop Interference (ms) |
| HIV Status (PWOH = ref)  PWH |  |  |  |  |  |
| Coefficient | 18.35 | -1.52 | -0.02 | 13.42 | 43.38 |
| 95% CI | [-4.84,41.54] | [-4.69, 1.65] | [-0.07, 0.02] | [-21.35,48.20] | [2.99,83.78] |
| p-value | 0.121 | 0.349 | 0.257 | 0.449 | 0.035 |
|  |  |  |  |  |  |
| Smoking Condition (smoking as usual = ref)  Abstinent |  |  |  |  |  |
| Coefficient | 9.99 | -2.72 | 0.01 | 15.38 | 0.02 |
| 95% CI | [-4.07,24.05] | [-4.81,-0.64] | [-0.01, 0.03] | [-1.41,32.16] | [-31.55,31.60] |
| p-value | 0.164 | 0.011 | 0.308 | 0.073 | 0.999 |
|  |  |  |  |  |  |
| HIV Status x Smoking Condition |  |  |  |  |  |
| Coefficient | -2.30 | 0.06 | -0.02 | 2.19 | -18.20 |
| 95% CI | [-24.00,19.40] | [-3.14, 3.26] | [-0.05, 0.01] | [-23.48,27.86] | [-66.36,29.96] |
| p-value | 0.836 | 0.972 | 0.266 | 0.867 | 0.459 |
|  |  |  |  |  |  |
| Lab Session Number (first = ref)  Second Lab session |  |  |  |  |  |
| Coefficient | -11.08 | -1.46 | 0.04 | 2.95 | -35.68 |
| 95% CI | [-21.82,-0.35] | [-3.04, 0.13] | [0.02, 0.05] | [-9.79,15.69] | [-59.60,-11.76] |
| p-value | 0.043 | 0.072 | 0.000 | 0.650 | 0.003 |
|  |  |  |  |  |  |
| Race (White = ref)  Black/African American |  |  |  |  |  |
| Coefficient | 2.26 | -2.49 | -0.00 | -6.92 | 30.16 |
| 95% CI | [-28.89,33.40] | [-6.57, 1.59] | [-0.06, 0.06] | [-55.13,41.29] | [-18.62,78.94] |
| p-value | 0.887 | 0.232 | 0.942 | 0.779 | 0.226 |
| Race (All others) |  |  |  |  |  |
| Coefficient | -22.91 | -4.60 | -0.01 | -39.81 | 43.13 |
| 95% CI | [-64.61,18.78] | [-10.24, 1.04] | [-0.09, 0.07] | [-106.39,26.78] | [-23.75,110.01] |
| p-value | 0.281 | 0.110 | 0.769 | 0.241 | 0.206 |
|  |  |  |  |  |  |
| Gender (Male = ref)  Female |  |  |  |  |  |
| Coefficient | 9.56 | 1.12 | 0.01 | -11.02 | 11.36 |
| 95% CI | [-9.91,29.03] | [-1.49, 3.72] | [-0.03, 0.05] | [-41.76,19.73] | [-19.60,42.33] |
| p-value | 0.336 | 0.400 | 0.683 | 0.482 | 0.472 |
|  |  |  |  |  |  |
| Nicotine Dependence (FTND) |  |  |  |  |  |
| Coefficient | 1.29 | -0.18 | -0.00 | 1.20 | 3.46 |
| 95% CI | [-3.77, 6.35] | [-0.86, 0.49] | [-0.01, 0.01] | [-6.77, 9.17] | [-4.57,11.49] |
| p-value | 0.617 | 0.600 | 0.971 | 0.768 | 0.398 |
|  |  |  |  |  |  |
| Age (years) |  |  |  |  |  |
| Coefficient | 1.14 | -0.15 | -0.00 | 2.10 | 1.22 |
| 95% CI | [0.18, 2.09] | [-0.28,-0.02] | [-0.00,-0.00] | [0.58, 3.63] | [-0.32, 2.76] |
| p-value | 0.019 | 0.025 | 0.004 | 0.007 | 0.121 |
|  |  |  |  |  |  |
| SILS |  |  |  |  |  |
| Coefficient | -0.32 | 0.32 | 0.00 | 1.23 | 1.12 |
| 95% CI | [-1.04, 0.40] | [0.22, 0.41] | [0.00, 0.01] | [0.09, 2.37] | [-0.02, 2.27] |
| p-value | 0.381 | 0.000 | 0.000 | 0.035 | 0.054 |
|  |  |  |  |  |  |
| Income(<$20,000 = ref)  >=$20,000 |  |  |  |  |  |
| Coefficient | -14.14 | -0.42 | 0.03 | 11.69 | -36.59 |
| 95% CI | [-34.39, 6.11] | [-3.11, 2.27] | [-0.01, 0.07] | [-20.13,43.50] | [-68.55,-4.63] |
| p-value | 0.171 | 0.758 | 0.100 | 0.472 | 0.025 |
|  |  |  |  |  |  |
